# Co-design and clinician evaluation of resources to address weight stigma in antenatal care

**DOI:** 10.1186/s12884-025-07327-3

**Published:** 2025-03-08

**Authors:** Briony Hill, Haimanot Hailu, Bec Jenkinson, Siarn Rakic, Taniya S. Nagpal, Jacqueline A. Boyle, Penelope Sheehan, Sarah Darlison, Helen Skouteris

**Affiliations:** 1 Health and Social Care Unit, School of Public Health and Preventive Medicine, Monash University, 553 St Kilda Road, Melbourne, VIC 3004 Australia; 2 Australian Women and Girls’ Health Research Centre, School of Public Health, The University of Queensland, Brisbane, Australia; 3 Faculty of Kinesiology, Sport, and Recreation, University of Alberta, Edmonton, Canada; 4 Health Systems and Equity, Eastern Health Clinical School, Monash University, Melbourne, Australia; 5 Director of Obstetrics, Eastern Health, Eastern Health, Melbourne, Australia; 6 Department of Obstetrics and Gynaecology, Monash University, Melbourne, Australia; 7 Warwick Business School, Coventry, UK

**Keywords:** Weight stigma, Maternity care, Co-design, Midwife, Lived experience

## Abstract

**Background:**

Weight stigma is a commonly reported experience in maternity care that negatively impacts the health of mothers and their babies. Knowledge to inform weight stigma reduction efforts in antenatal care is urgently required. This study aimed to co-design weight stigma reduction resources in antenatal care and evaluate clinician perspectives of the resources regarding their relevance to practice, strengths, and areas for improvement.

**Methods:**

We conducted a five-phase co-design project involving consumers (*n* = 8) and clinicians (midwives *n* = 16, obstetrician *n* = 1), with outputs from each stage informing the next: (1) engaging with key stakeholders; (2) prioritising the voices of lived experience through a consumer stories video; (3) three co-design workshops to inform resource development; (4) resource production; and (5) qualitative evaluation of the resources. The co-developed resources were evaluated via interview where clinicians viewed or listened to the resources and described their engagement and satisfaction with the resources, their relevance to practice, and perspectives on the strengths, areas for improvement, and feasibility for achieving the resources’ intended goal. Transcripts were analysed using descriptive thematic analysis.

**Results:**

We produced a set of evidence-based resources co-designed by consumers and clinicians including a consumer video designed to elicit empathy about lived experiences of weight stigma in maternity care, images representing women with diverse body sizes for use in clinic waiting rooms, a short podcast to raise awareness of weight stigma in maternity care, and signposts for the antenatal clinic to prompt clinicians to consider weight stigma in everyday clinical interactions. Clinicians who saw the resources reported that they were valuable and relevant to practice and were important and helpful introductory materials to the issue of weight stigma. Pragmatic examples of reducing weight stigma in clinical interactions were requested.

**Conclusions:**

Maternity care clinicians have an appetite to improve their learning opportunities to tackle weight stigma in practice. Further refinement of the resources, evaluation of the effectiveness at changing clinician behaviour, and implementation into health services are logical next steps. Reducing women’s experiences of weight stigma should lead to better care and better pregnancy outcomes for larger bodied women.

**Clinical trial number:**

Not applicable.

**Supplementary Information:**

The online version contains supplementary material available at 10.1186/s12884-025-07327-3.

## Background

Pregnancy necessitates frequent healthcare visits and interactions with healthcare providers and, problematically, the healthcare setting is one of the most common sources of weight stigmatisation [1]. Weight stigma is defined as attributing negative stereotypes and prejudiced attitudes towards individuals based on body weight [2]. Several studies have documented the pervasiveness of weight stigma in healthcare settings [1, 3, 4]. A survey conducted with a large sample of medical students in the US revealed that 74% harboured implicit weight bias, while 67% reported holding explicit weight bias [5]. Larger bodied women (often defined as women living with obesity in the literature) [6] identified healthcare as one of the common sources of their weight stigmatisation experiences, reporting feelings of judgment, guilt, and shame about their weight during healthcare visits [1]. An Australian study found that maternity care providers had less favourable attitudes towards caring for women with larger bodies compared to those of women in the “normal weight” body mass index category [3]. Weight stigma leads to poorer mental and behavioural health, decreased access to and uptake of reproductive healthcare, and poorer health outcomes in pregnancy, birth, and longer-term maternal-child health [7]. Given the substantial evidence indicating the pervasiveness of weight stigma in health care and its negative health impacts, eliminating weight stigma is essential to improving women’s pregnancy outcomes [8, 9].

A 2022 international transdisciplinary co-design workshop that aimed to understand why weight stigma occurs and how it can be addressed for reproductive aged women identified empowering and equipping healthcare professionals (HCPs) to address weight stigma in their practice as one of the key aspects of working towards stigma elimination [10]. Also highlighted was the importance of incorporating lived experience perspectives, and recommended strategies education and information about weight bias and its impacts [10]. Similarly, a recent systematic review identified that effective interventions for reducing weight stigma in healthcare incorporated increasing education about weight-related health, the cause and controllability of obesity, and empathy-evoking activities that enhance understanding of the lived experiences of people living with obesity [11]. The review also found a weight-inclusive approach and combined weight stigma reduction strategies to help improve the attitudes of HCPs towards people living with obesity [11].

Despite increasing evidence to guide intervention development to address weight stigma, there is a notable absence of targeted interventions to address this issue within maternity care. Excluding maternity care from weight stigma reduction efforts may have serious intergenerational implications and exacerbate health inequalities [12]. Furthermore, considerations of weight stigma in maternity care are unique because it is about optimising care provision and maternal and infant outcomes; it is not about obesity prevention or treatment [7]. Knowledge to inform weight stigma reduction efforts in antenatal care is urgently required for rapid policy and practice change.

In the limited interventions to address weight stigma in general, a lived experience element is rarely considered [13]. Consumer and community involvement (CCI), sometimes also referred to as patient and public involvement or lived experience [14], involves actively partnering with those impacted by the research, rather than conducting research on their behalf [15]. Involving lived experience experts helps to create new knowledge (that is, lived experience evidence [13]) based on practical insights and individual needs that can be translated effectively to ensure interventions are feasible and relevant for those they are intended for [14]. Lived experience experts can be involved across all levels of research from problem identification and priority setting to research co-design and translation to practice [15, 16]. Co-design methodology involves people who are likely to be impacted by or will benefit from the process and/or the outcome, either directly or indirectly, to ensure the final solution better meets their needs [17, 18]. The principles of successful co-design include creating equal partnerships with lived experience experts, working on a shared goal, trust, acknowledging and valuing their views and experiences, practicing empathy, and maintaining a safe environment [19]. Co-design is particularly useful in implementation science when the outcomes of the process influence the practice and outcomes of those involved [14, 20]. To our knowledge, no previous interventions addressing weight stigma have incorporated co-design methods. Therefore, in this study, we aimed to co-design weight stigma reduction resources in antenatal care and evaluate clinician perspectives of the resources regarding their relevance to practice, strengths, and areas for improvement.

## Methods

**Fig. 1:**
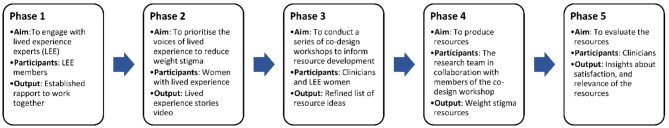
Phases of weight stigma resources co-design

We conducted a five-phase co-design project involving lived experience expert participants (i.e., women with lived experience of weight stigma in maternity care) and clinicians, with findings from each stage informing the next (Fig. 1). Ethical approval was obtained through the Eastern Health Human Research Ethics Committee (LR23-015-92443) and the Monash University Human Research Ethics Committee (36497 and 37944). Informed consent was obtained from both lived experience expert participants and clinicians for all phases of the research. The investigator team comprised academics with expertise in weight stigma, consumer advocacy and engagement, co-design, participatory research, implementation science, health psychology, qualitative research methods, public health and health promotion, reproductive and women’s health, as well clinical expertise in obstetrics and gynaecology. The research was conducted between January 2023 and May 2024. As the project was focused on developing rather than delivering an intervention, it was not registered in a clinical trials registry.

### Phase 1: stakeholder engagement and participant recruitment

We engaged with lived experience experts via our Body Positive Birth Lived Experience Expert (LEE) group. The LEE group comprised of nine women who responded to social media posts shared by Australian maternity consumer organisations seeking women who were concerned that their body size had impacted on their maternity care. These women initially took part in informal discussion groups with members of the research team, and expressed interest in ongoing involvement in research. Based on our established relationships with the LEE group and our knowledge of their confidence and capacity to speak publicly about their experiences, we invited two women to engage with the project team on a consumer stories video (Phase 2). Of these two women, one was a First Nations woman while the other did not disclose her ethnicity, and both women lived in the metropolitan region of a large Australian city. In addition, we also invited all LEE members to participate in the resource development phase of the project (Phase 3); six women agreed to take part (66.6%). The women were remunerated for their time and knowledge in the form of “gift pay” gift cards per Monash University’s gift card policy.

Clinicians (midwives and an obstetrician) were involved in Phases 3 and 5 of the research; they were recruited from a large metropolitan health service in Melbourne, Victoria, via email invitations from their department head to participate in the research immediately prior to the commencement of each phase of the project. The health service covers more than 775,000 people in its primary catchment area and provides its service from more than sixty different locations. The maternity care clinic provided pregnancy, birth, and postnatal care services. Furthermore, for Phase 5, researchers visited the health service to explain the study in person and were available to answer any questions. Three midwives agreed to participate in Phase 3 and 10 in Phase 5; one obstetrician participated in Phase 5. There were no exclusion criteria for clinician participation. In total, eight consumers and 17 clinicians (midwives *n* = 16, obstetrician *n* = 1) took part.

### Phase 2: prioritising the voices of lived experience – consumer stories video

As a result of the LEE engagement during project conceptualisation, a consumer stories video was planned as part of the project. The two consumers were interviewed separately by a member of the research team (BJ) who had a strong working relationship with the women and is an expert in consumer advocacy. The interview was designed to elicit a natural account of the women’s experiences of weight stigma in maternity care and what was important to them to share in the video. The interview transcript was then reviewed by two members of the team (BJ and BH) and excerpts were identified to be cut into shorter audio snippets. A video designer cut the interview audio together and this was sent to the consumers for their approval. Finally, the video designer assembled the video together with carefully selected audio, imagery, and music.

Ultimately, a two-minute 50 s video was created encapsulating the two women’s stories of their lived experiences of weight stigma in maternity care. The video included: (1) a short introduction by an obstetric physician about weight stigma; and (2) the two women’s personal stories of their experiences, including positive and negative encounters in relation to weight during their maternity care. Imagery from the women’s personal collections, stock images, and graphically designed images were included alongside key text points and emotive music. Closed captions were added to the video to increase accessibility. This video was short in length to increase engagement. The video was designed to elicit empathy from the watcher, as an evidence-based strategy to address weight stigma attitudes and beliefs [11].

### Phase 3: resource development – co-design workshops

The aim of Phase 3 was to co-design educational resources to reduce weight stigma in the antenatal care setting. The co-design group (six consumers and three midwives) participated in three one-hour Zoom workshops to co-design the resources. The workshops were facilitated by two researchers with experience in program facilitation, research, engagement, and weight stigma (BH and HH). A consumer advocate (SR) also attended the workshops to minimise any potential power imbalances introduced by the inclusion of both women and midwives together. However, the consumer advocate was rarely utilised, with the women confidently contributing to the discussions. Workshop activities included presentations from the researchers on the importance of developing the resources, peer-reviewed evidence to identify a “menu of options” that could contribute to resource development [11, 21], and activities to list and prioritise topics and identify potential resource delivery methods. All workshops were interactive with the key goal to generate LEE participant- and midwife-led ideas to progress the resource development. Large and small group activities were included. Workshop activities included blue-sky thinking (workshop 1) [22], star bursting (workshops 2 and 3) [23], and the New-Useful-Feasible matrix (workshop 3) [24].

From the blue-sky thinking in the first workshop, we distilled the key areas that were priorities for additional focus within the next two workshops. Blue-sky thinking involves brainstorming without limitations or thinking outside of the box for creation of new ideas [22]. The three areas generated by the co-design group were: (1) representation of women in larger bodies; (2) high risk assumptions in pregnancy based solely on body size; and (3) addressing empathy and education in healthcare professionals. The participants also felt that addressing policies, particularly hospital policies that were stigmatising and removed women’s agency in relation to their care, was a significant priority. Through group discussion, it was agreed that policy change was outside the scope of this project but should be a focus of future work. The key priorities were included in a star bursting activity. Star bursting involves identifying the who, what, when, where, why, and how of your topic on the tips of a 6-pointed star [23]. The co-design group applied the star bursting method to each of the three priority areas on collaborative online whiteboards using the online platform, Mural [25].

Using the workshop audio recordings and starburst Mural boards and applying a descriptive thematic approach, the researchers distilled the co-design groups’ priorities for resource development into several key activities: (1) displaying happy, healthy larger-bodied women on all resources produced; (2) developing long (e.g., 1–2 days) in-service weight stigma training for staff; (3) developing short, interactive activities for staff such as lunch and learns, podcasts, or reflective activities; (4) distributing flyers, posters, and email communications about weight stigma; (5) developing educational learning modules; and (6) developing resources to guide clinical encounters such as sign-posts.

The New-Useful-Feasible (NUF) matrix activity (workshop 3) enabled further distillation of the six ideas produced through the star bursting into a short list of activities. The NUF matrix is a useful tool to identify ideas that have been selected and are likely to be effective, implementable, and work in practice [24]. All members of the co-design group answered three questions per activity suggestion, scoring them based on whether they were *new* to the setting, perceived as *useful* for HCPs, and how *feasible* they were to implement, using a rating score from 0 to 10 (10 being extremely new/useful/feasible) for each question [24]. With three questions per activity, possible scores could range between zero and 30. The means, standard deviations, and range of scores across the suggested activities are provided in Table 1 (combined for midwives and consumers due to the small sample size). The patterns of scores were similar across both midwives and consumers. While all activities scored relatively highly, the top three were selected for further development based on available resources and time constraints. These were the representation of larger-bodied women on resources; short, interactive activities; and resources guiding clinical consultations.

**Table 1:** New-useful-feasible (NUF) matrix scores for suggested activities

Suggested activity/resource	NUF score	
	**Mean (SD)**	**Range**
Happy, healthy larger-bodied women represented on all resources produced*	28.1 (1.6)	26–30
Long (e.g., 1–2 days) in-house weight stigma training	22.5 (9.0)	6–30
Short, interactive, activities*	26.3 (3.2)	21–30
Flyers, posters, and email communications about the topic	23.6 (6.4)	10–30
Educational modules	23.5 (4.6)	17–30
Resources guiding clinical consultations*	25.5 (3.7)	19–30

*activities selected for further development

### Phase 4: resource production

We used an iterative process for the resource development that incorporated multiple rounds of feedback from both the investigator team and the co-design workshop participants. Firstly, two investigators (BH and HH) reviewed the workshop ideas, recordings, and transcripts to glean practical ideas to embody the suggested resource activities that were prioritised in the workshops. Second, these ideas were discussed with two obstetrician/gynaecologist-researchers who were also part of the investigator team (JB, PS) to identify what would be practical to implement in the clinic setting. Third, the refined ideas, including the topic, text, or image choices for the resources, were presented to both the investigator team and co-design group concurrently and further improved based on their feedback. Finally, the resources were developed along with the notes on the context within which they might be implemented and circulated to the entire investigator team and co-design group for their final input.

The resources developed included: (1) images of pregnant women for the digital monitor screen in the clinic waiting room; (2) a short podcast to raise awareness of weight stigma in maternity care; and (3) signposts for the antenatal clinic to prompt clinicians to consider weight stigma in their clinical interactions. Examples of resources 1 and 2 are displayed in Fig. 1a and b. All resources developed are provided in the supplementary file (Figures S1a-f; Figures S2a-d; Table S1). The images were professional photographs taken of a pregnant woman towards the end of her pregnancy who self-identified as larger bodied. As per the recommendations made by women in the workshop, the photographs aimed to show that women of any body size can be happy and healthy. The podcast included a midwife interviewing a researcher with expertise in weight stigma. It covered topics including what weight stigma is, how common it is, the consequences of weight stigma, examples of weight stigma in maternity care, and ways healthcare professionals can reduce weight stigma in maternity care settings. The signposts were created by the research team, with substantial input from consumers and midwives to refine the wording. The signposts were about postcard size and intended to sit in a visible place in the clinic room such as at the corner of the computer monitor, or above the weighing scales. They included wording such as, “respect and dignity at every size” and “the language you use will last a lifetime”.

### Phase 5: resource evaluation

The aim of Phase 5 was to evaluate the feasibility of the resources that were produced in Phases 2 and 3. Ten midwives and one obstetrician, not part of the co-design group, participated in a Zoom interview (mean duration 21 min, standard deviation 4.9 min; range 14–28 min) with a researcher (HH). During the interview, participants were invited to look at or listen to the resources co-developed during the workshops. The empathy video (Phase 2) was not included as this video had already undergone an extensive consultation process and approval by the two LEE women who were featured. Following review of the resources, clinicians filled out an evaluation checklist and responded to semi-structured questions. The evaluation checklist was informed by the New World Kirkpatrick Model [26] and included questions on the HCP’s engagement and satisfaction with the resources and their relevance to practice. The qualitative interview guide (see supplementary file Table S2) was used to gain an understanding of the clinicians’ perspectives on the strengths and areas for improvement, relevance, and feasibility for achieving the resources’ intended goal.

The interview recordings were transcribed verbatim and analysed using descriptive thematic analysis. Transcripts were coded with two lenses. Firstly, consistent with the objectives of the study, descriptive themes associated with the study aims were identified a priori (e.g., relevance and strengths of the resources). Secondly, the interviews were analysed inductively by HH to generate any previously unforeseen themes. Accordingly, three themes were identified and refined through discussion, initially between HH and BH and then via consultation with all authors. NVivo software was used to code all transcripts.

## Results

**Fig. 2:**
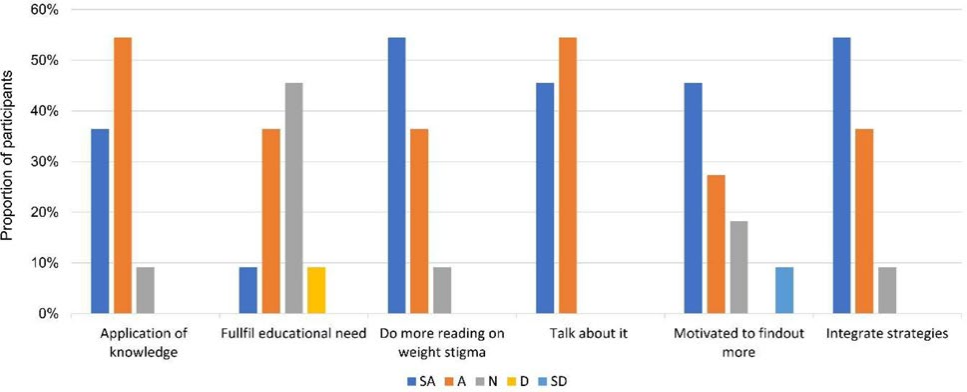
Clinician engagement, satisfaction with the resources, and relevance to their practice informed by level 1 Kirkpatrick’s model; SA: Strongly Agree, A: Agree, N: Neutral, D: Disagree, SD: Strongly Disagree

Findings for Phase 5 are presented below. Participant characteristics are shown in Table 2. All participants were female and worked as antenatal clinicians for an average of 4.8 years (SD = 3.5). Clinicians, who had not been involved in the resource development, rated their engagement, satisfaction with the resources, and relevance to their practice on five-point Likert scale (Fig. 2). Overall, clinician satisfaction was positive and indicated that the resources prompted HCPs to do further reading or seek more educational resources on the topic of weight stigma, integrate the strategies into their practice, and talk to colleagues about weight stigma. Of note, the resources did not completely fulfil the educational needs on weight stigma in pregnancy for six of the 11 participants, however, nine clinicians rated the quality of the resources as very good or excellent.

**Table 2:** Clinician characteristics for the resource evaluation (phase 5)

Variable	Categories	Frequency *N* (%)
Gender	Female	11 (100)
Professional role	Midwife	10 (90.9%)
	Obstetrician	1 (9.1%)
Service as antenatal care clinician	1–2 years	4 (36.4%)
	3–5 years	4 (36.4%)
	6 years and above	3 (27.3%)
	Mean, years (SD)	4.8 (3.5)
Prior familiarity with the issue of weight stigma in antenatal care	Yes	10 (9.1%)
	Somewhat	1 (90.9%)
	No	0

### Qualitative findings

Overall, we identified two themes: Resources (partially) address clinician’s unmet need for guidance on avoiding weight stigma; and suggestions to optimise effective implementation of the resources. Illustrative quotes are presented in Table 3.

**Table 3:** Phase 5 resource evaluation themes with illustrative quotes

Themes	Subthemes	Selected illustrative quotes
Theme 1: *Resources (partially) address clinician’s unmet need for guidance on avoiding weight stigma*	Subtheme 1: The resources are relevant to clinical practice and enhance awareness of weight stigma	*“I think it’s [the resources] very relevant. I think that weight stigma is not really well understood or discussed in the context of antenatal care. And I think any resources would contribute to an improvement in that.”* Clinician 2, midwife *“I do think everybody cares really deeply about the women that they’re looking at…and do not want to do any harm. So*,* I think the resources could definitely motivate people to think carefully about what they say.”* Clinician 1, midwife *“I think the podcast got more information specifically about stigma*,* and challenges health care providers views and makes them reflect on their own practice.”* Clinician 3, obstetrician *“I think just being reminded of these sorts of things [weight stigma] is a really good prompt to making sure that we’re practicing with the best information in mind.”* Clinician 11, midwife *“I think*,* the photos and the signage are very visual*,* so one can get the message through in a fleeting glance*,* you know*,* you want to catch people’s attention at any point.”* Clinician 4, midwife
	Subtheme 2: Suggestions for the resources to be more informative	*“…we never intentionally set out to hurt someone. We’re only doing it from a place of care… so having*,* little phrases or wording that we could say [non-stigmatising] would be probably helpful….” Clinician 8*,* midwife* *“I don’t think they need to be improved. I think they’re really good.”* Clinician 6, midwife
Theme 2: Suggestions to facilitate effective implementation of the resources	-	*“I do think that getting champions within a workplace that are well respected*,* and that are clinicians of different backgrounds would be helpful*,* you know to advocate for a shift in the way that we approach these things.”* Clinician 2, midwife *“You could make it like a part of a learning package*,* you know*,* how we’ve got to do our compulsory learning every year. You could have something like that or put it in a booklet.”* Clinician 4, midwife

### Theme 1: resources (partially) address clinicians’ unmet need for guidance on avoiding weight stigma

This theme describes ideas related to the relevance, strength, and areas of improvement for the resources. This was captured across two subthemes: the resources are relevant to clinical practice and enhance awareness of weight stigma; and suggestions for the resources to be more informative.

### Subtheme 1: the resources are relevant to clinical practice and enhance awareness of weight stigma

The clinicians mentioned that weight stigma is not well understood or discussed in the context of existing antenatal care. The clinicians also felt that any resources that targeted weight stigma would be very relevant given the current scarcity of such resources. They reported that the resources would be helpful to achieve goals of reducing weight stigma in antenatal care practice if used as introductory materials to the concept of weight stigma. The clinicians also mentioned that these resources would be helpful to start challenging existing weight stigma norms and bring the issue to the front of mind. The clinicians felt that these resources would motivate them to reflect and avoid unintended harm, as they deeply care about their patients.

The clinicians mentioned several *strengths* for the resources they were presented with. Firstly, clinicians reported that the resources enhanced awareness of weight stigma based on scientific evidence. Specifically, the participants described that the provision of information on the negative consequences of weight stigma (i.e., in the podcast) and what can be done to reduce weight stigma during clinical practice was pragmatic and helpful. Of a similar vein, the clinicians also mentioned that one of the positive sides of the podcast was elaborating on the actual risk of complications for women living in larger bodies based on evidence. They highlighted that clinical practice tends to be risk averse and most hospital policies and guidelines already label larger bodied women as ‘high risk’; the clinicians felt that these resources will help to put those sorts of risks in perspective.

A second strength of the resources was that they promoted normalisation and representation of larger-bodied women, adding that this would be helpful for women to see these images as it makes them feel welcomed. A third strength was that the resources acted as a reminder to be mindful of language and break down biases. This was specifically mentioned in terms of having respectful conversations with women as well as to help clinicians reflect on their practice. A fourth strength of the visual resources was that they appealed as a way of catching clinicians’ attention by using clear and simple imagery and or text.

### Subtheme 2: suggestions for the resources to be more informative

Notwithstanding the strengths, areas for improvement identified included suggestions for the resources to be more informative. This was not a universal recommendation and others felt that the resources did not need any improvement. Clinicians specifically mentioned it would be helpful to include examples of suitable non-stigmatising phrases or language that could be used by clinicians in practice. The participants also felt that the images displayed in the resources lacked representation of diverse body weights and shapes (noting that only one woman was photographed). A further suggestion for improvement for the signpost was to consider frequently updating the signpost to prevent clinicians becoming desensitised to its presence.

### Theme 2: suggestions to facilitate effective implementation of the resources

The clinicians offered key suggestions such as appointing a workplace champion to take responsibility for the resources; making structural changes to the healthcare environment, such as weight inclusive infrastructure, culture, and guidelines, so that efforts to reduce weight stigma are not undermined; developing resources for patients; broadly advertising the resources to ensure they reach the target audience; making the resources part of a mandatory learning package; and availing the resources to HCPs beyond antenatal care.

## Discussion

This project aimed to co-design resources to address weight stigma in maternity care and evaluate clinicians’ perspectives of the resources regarding their relevance to practice, strengths, and areas for improvement. To our knowledge, this is the first set of resources co-designed to address weight stigma in maternity care. We conducted a five-phase process incorporating engagement of women with lived experience, midwives, and an obstetrician; co-production of resources; and evaluation of the resources. The outcome was a set of evidence-based resources that were seen by clinicians to be valuable and relevant to practice. Resources included a consumer video designed to elicit empathy about lived experiences of weight stigma in maternity care, images representing women with diverse body sizes for use in clinic waiting rooms, a short podcast to raise awareness of weight stigma in maternity care, and signposts for the antenatal clinic to prompt clinicians to consider weight stigma in every clinical interaction. Clinicians also volunteered suggestions for next steps to optimise implementation of the resources into practice.

Through the co-design workshops we identified that stigma reduction resources should fulfil specific needs to be useful in practice. These included being able to be integrated into practice (i.e., the signposts), acting as consistent reminders to be size-friendly (i.e., the representation of diverse body sizes), and meeting knowledge gaps (i.e., the podcast). These resources complement recommendations from a review to eliminate weight stigma in antenatal healthcare settings [27]. The review authors identified sensitivity training for discussions around weight, promoting a patient-centred approach, maintaining open communication when discussing risks with women, and improving referral practices [27]. Our participants identified pragmatic ways to apply new knowledge, which may make them appealing and therefore easily implementable. The resources were also “low hanging fruit”, as they were low cost from both a development perspective and in terms of time needed to engage with them. It is well established that educational interventions are an effective and relevant component in improving HCP performance and therefore patient health outcomes [13, 28].

Our findings highlight an appetite for practical and user-friendly resources to help address weight stigma in maternity care. Throughout the workshops and interviews, it was clear that the clinicians wanted to provide non-stigmatising woman-centred care. Women and midwives perceive woman-centred care to include themes such as respect, partnership in decision making, promoting women’s autonomy, individualising care, and ensuring staff competency [29]. There are clear parallels between these factors and care that is not weight stigmatising. Indeed, clinicians are increasingly seeking out ways to recognise and eliminate weight stigma in their practice [11, 30, 31]. However, barriers remain such as structural factors that drive weight stigma and clinicians’ own unrecognised or implicit biases [3, 32]. Our use of co-design processes that integrated lived experience with clinician perspectives may have enabled implicit biases to be realised and hence addressed. Certainly, the midwives and lived experience consumers participated enthusiastically and harmoniously in the workshops. However, future research would need to explicitly address and answer this hypothesis.

Despite the positive feedback about the resources, several opportunities for their continual refinement were identified during the interviews, including more examples of implementable suggestions such as ways to have non-stigmatising conversations with women. This clinician education could be achieved with the Healthy Conversations Skills (HCS)© technique. Healthy Conversations Skills uses empowering, exploratory conversations in which health professionals engage and support individuals to make decisions about their behaviours and health care [33]. Healthy Conversation Skills have been predominately used as a way of discussing nutrition, physical activity, and weight gain in maternity care [34]. However, it has potential for application in weight stigma through its coaching style and non-judgemental approach to behaviour change, thereby addressing clinician behaviour, rather than the behaviour of women seeking care. Future research could explore the potential of HCS in reducing weight stigmatising experiences of women in maternity care.

Additional feedback received on the resources included ensuring broad diversity of body sizes was represented in the images, as well as diversity in other characteristics. This feedback was duly noted as it helps to enhance representation of women with diverse characteristics; due to time and financial constraints, we found it difficult to recruit pregnant women for the photos, especially women with larger bodies and representing diverse backgrounds. This resulted in much fewer images that we would have liked to use, as well as limiting the diversity that would better represent women of varied body sizes, ethnicity, and physical characteristics. This issue can be addressed in continual resource development before implementation.

The logical next step for this program of work is to refine the resources and develop an implementation plan for their integration into clinical practice. Clinicians volunteered some valuable suggestions to support this step, including appointing a project champion and integrating the resources into broader education around weight stigma. These suggestions align well with implementation science principles outlined in frameworks such as the Expert Recommendations for Implementing Change (ERIC) guidance [35]. The ERIC compilation provides distinct implementation strategies that can be utilised individually or combination in implementation research and practice [35]. Indeed, our broader co-design approach addressed some key steps for the early development of interventions to be implemented into health services and practice. These included involving people with lived experience and developing educational materials, making the first steps in implementation already achieved [35].

### Limitations and strengths

The limitations of this study included a lack of diversity of participants in terms of professional roles (i.e. clinicians) and gender. All of our participants were women and we had difficulty finding obstetricians/gynaecologists to participate; however, we captured this perspective by the inclusion of an obstetrician and a gynaecologist as investigators. Future co-design research could also consider bringing a broader array of voices to the process, including social workers, Aboriginal support workers, women’s support persons, and hospital administrative staff. Given the nature of the study, there is also a possibility of self-selection bias because the clinicians who agreed to participate might have an interest in the phenomenon of weight stigma. Given the adverse outcomes associated with weight stigma in maternity care [3, 7], this is potentially a benefit rather than a limitation. Lastly, even though the empathy video was not evaluated during phase 5 due to extensive consultation and approval by LEE women, future studies can measure changes in empathy as a result of watching the video, as well as evaluate whether any potential changes in empathy are associated with weight stigma attitudes, beliefs, or behaviours.

The strengths of this study lie in the fact that the resources were rigorously developed and evidence-based. They were also co-designed bringing in the perspectives of women with lived experience of weight stigma in maternity care and clinicians who the resources were designed for. Given clinicians’ reflections about the resources appeared to marry well with the goals of the resources it is likely they achieve their intended purpose.

## Conclusion

**Fig. 3:**
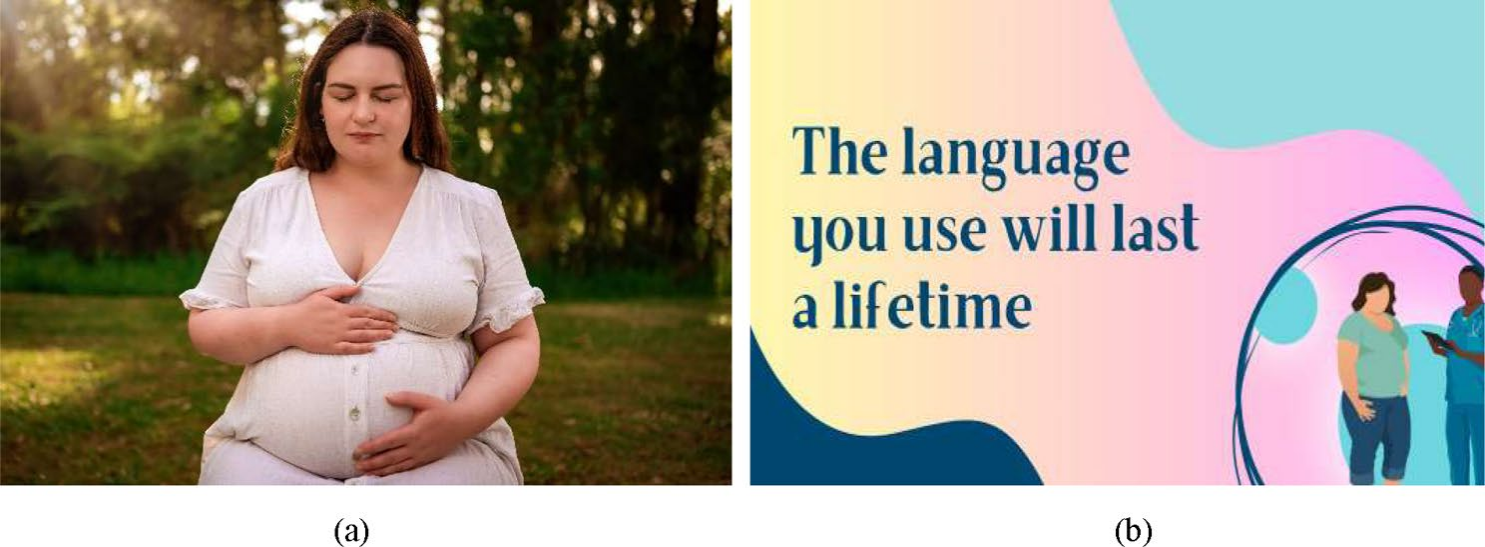
**a** image of pregnant woman for the clinic waiting room. **b** Signpost

Together with women with lived experience and clinicians, we have developed what we believe to be the first co-designed resources to address weight stigma in maternity care. The resources were positively evaluated by clinicians to be valuable and relevant to their practice. Hence, there is an appetite for midwives and obstetricians to improve their learning opportunities to reduce weight stigma in antenatal care and tackle weight stigma in practice. Future research should continue to refine the resources, evaluate their effectiveness at changing clinician behaviour, and implement them fully into health services. Doing so may reduce women’s experiences of weight stigma, which should lead to better care and better pregnancy outcomes for larger bodied women.

## Electronic supplementary material

Below is the link to the electronic supplementary material.

Supplementary Material 1

## Data Availability

Data analysed in this study are not publicly available because the participants have not consented to sharing their discussions and interviews, and to protect their privacy.
