# Supplementary material for: Co-design and clinician evaluation of resources to address weight stigma in antenatal care
Source: BMC Pregnancy Childbirth. 2025 Mar 8;25:263. doi: 10.1186/s12884-025-07327-3 (PMC11890724; doi:10.1186/s12884-025-07327-3)
Supplement: Supplementary file 1 — Supplementary Material 1 [file 12884_2025_7327_MOESM1_ESM.docx]

**Co-design and clinician evaluation of resources to address weight stigma in antenatal care**

Briony Hill ^1*^, Haimanot Hailu ^1^, Bec Jenkinson ^2^, Siarn Rakic ^1^, Taniya S. Nagpal ^3^, Jacqueline A Boyle ^4^, Penelope Sheehan ^5,6^, Sarah Darlison ^5^, and Helen Skouteris ^1,7^

^1^ Health and Social Care Unit, School of Public Health and Preventive Medicine, Monash

University, Melbourne, Australia

^2^ Australian Women and Girls’ Health Research Centre, School of Public Health, The

University of Queensland, Brisbane, Australia

^3^ Faculty of Kinesiology, Sport, and Recreation, University of Alberta, Edmonton, Canada

^4^ Health Systems and Equity, Eastern Health Clinical School, Monash University, Melbourne, Australia

^5^ Director of Obstetrics, Eastern Health, Eastern Health, Melbourne, Australia

^6^Department of Obstetrics and Gynaecology, Monash University, Melbourne, Australia

^7^ Warwick Business School, Coventry, UK

*Corresponding author: Dr Briony Hill, Health and Social Care Unit, School of Public Health and Preventive Medicine, Monash University, 553 St Kilda Road, Melbourne VIC 3004, Australia

Email: briony.hill@monash.edu

**Supplementary files**

**Resources co-designed to reduce weight stigma in maternity care**

**
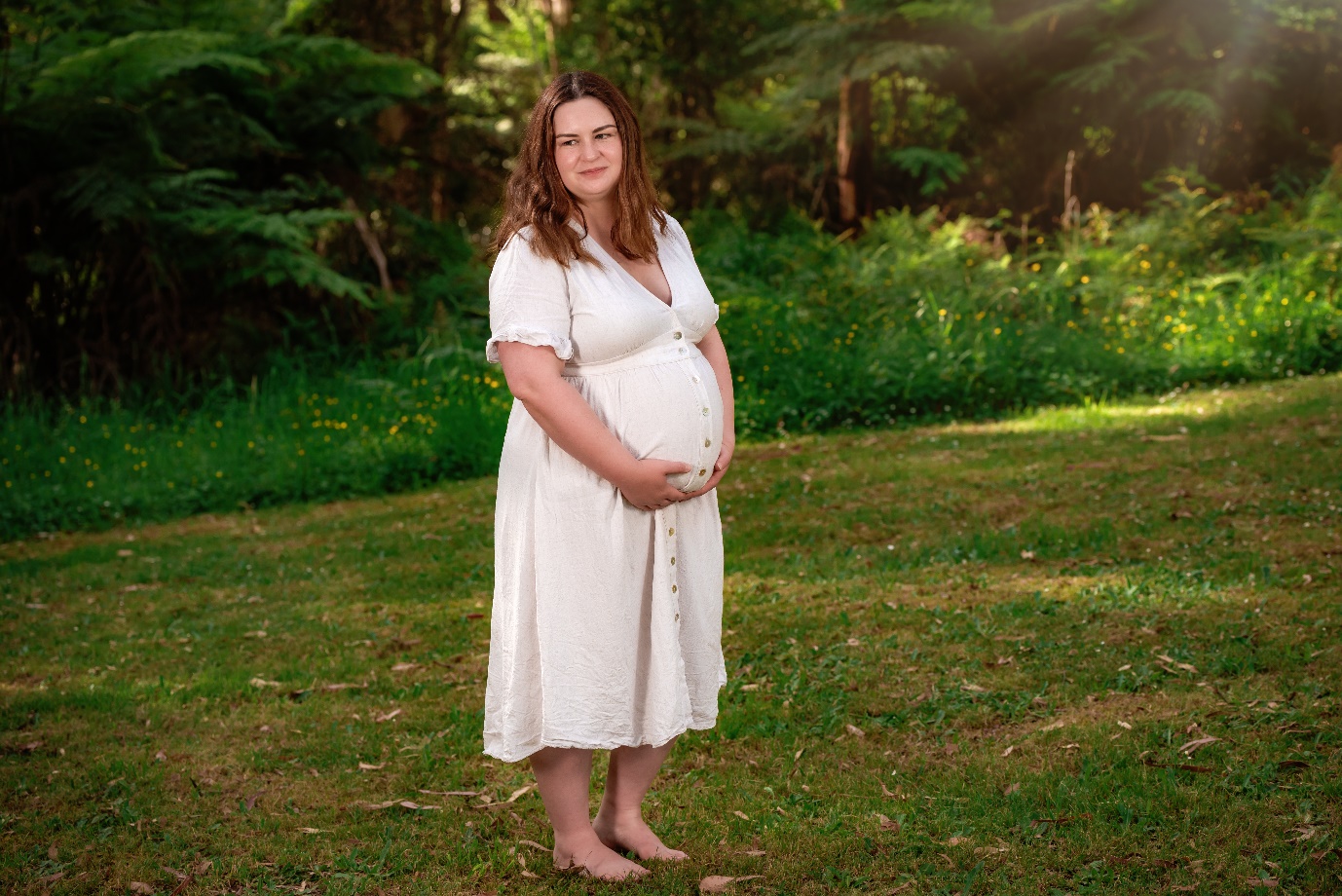
**

**Figure S1a:** Photo of pregnant woman for the digital monitor screen in the clinic waiting room

**
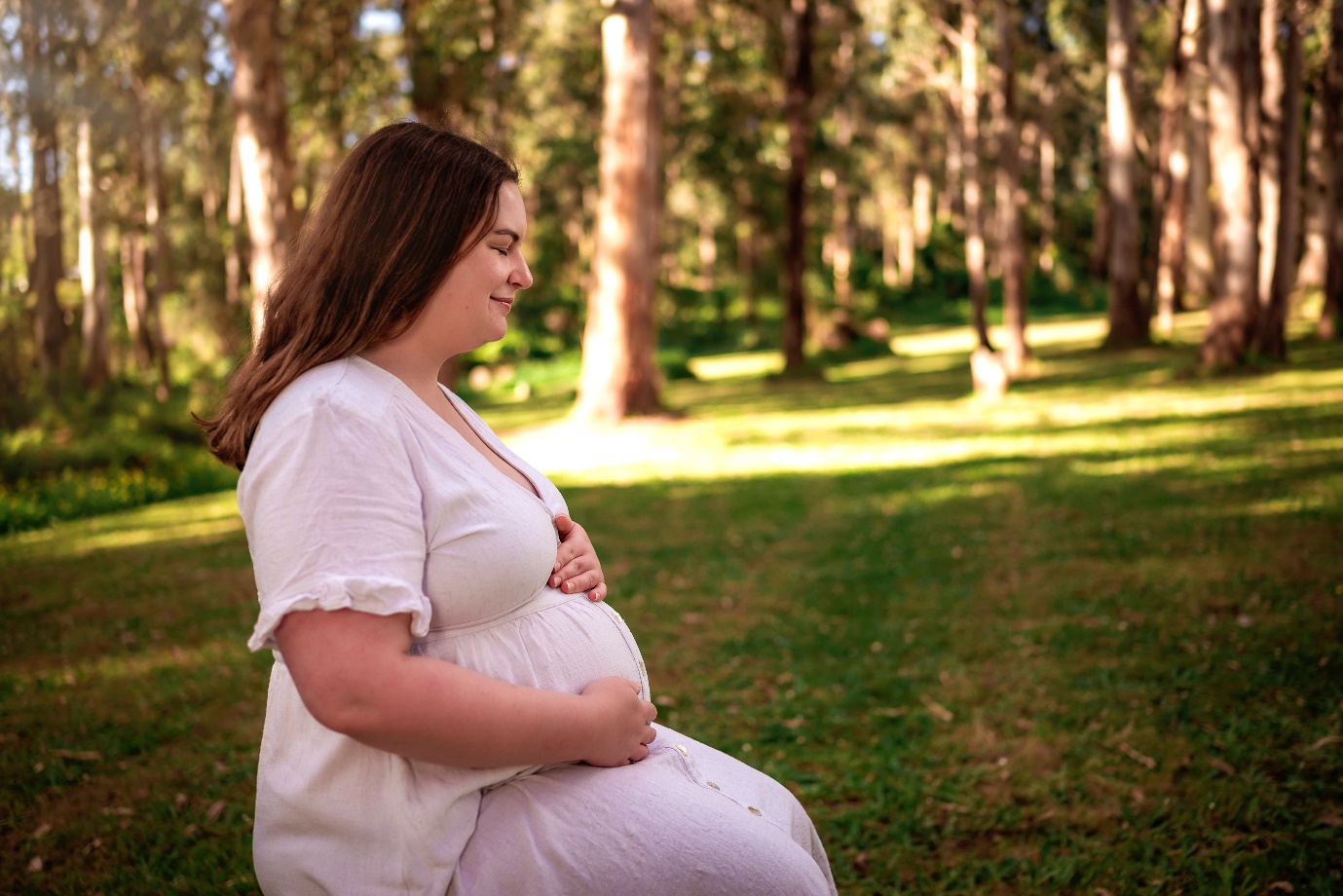
**

**Figure S1b:** Photo of pregnant woman for the digital monitor screen in the clinic waiting room

**
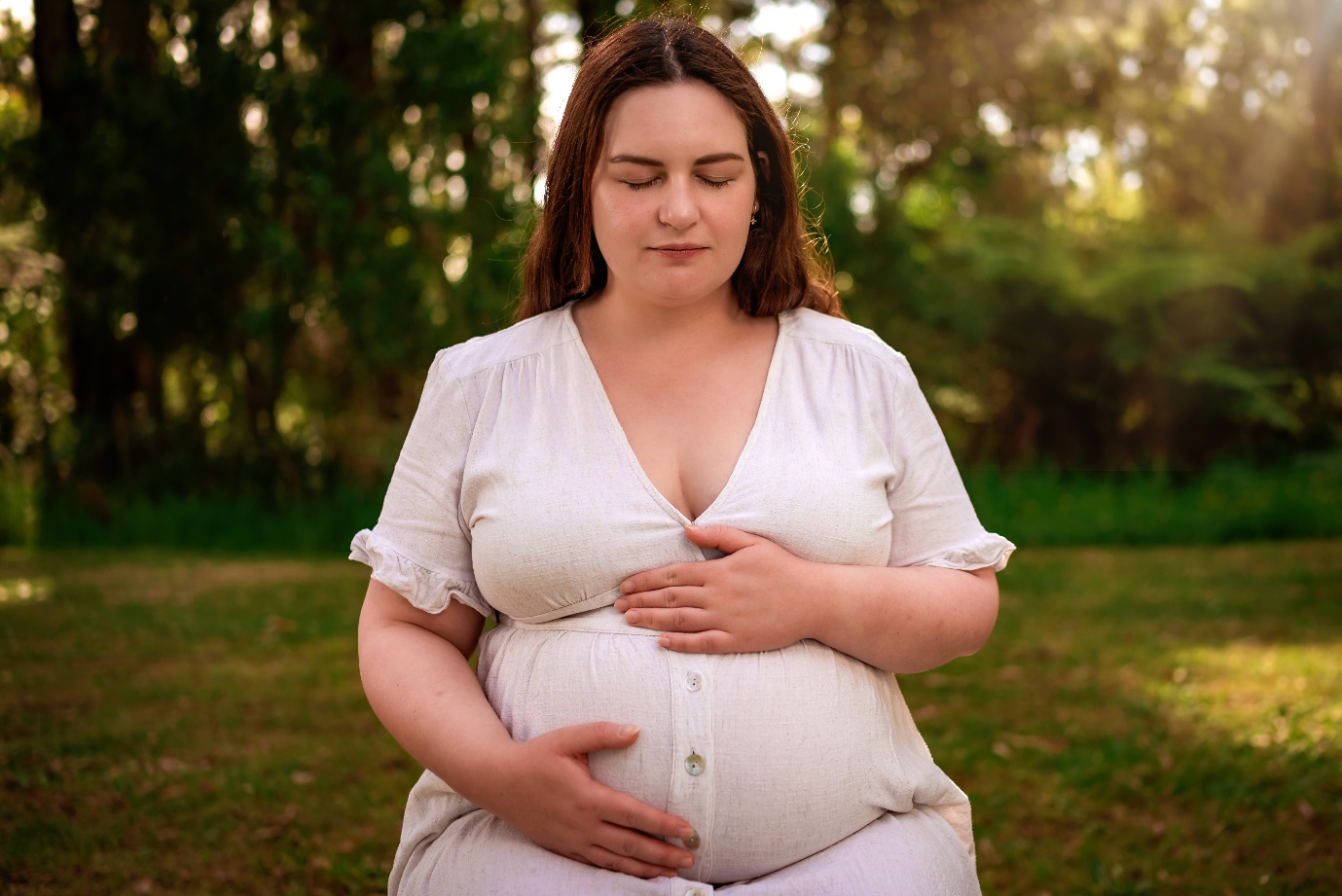
**

**Figure S1c:** Photo of pregnant woman for the digital monitor screen in the clinic waiting room

**
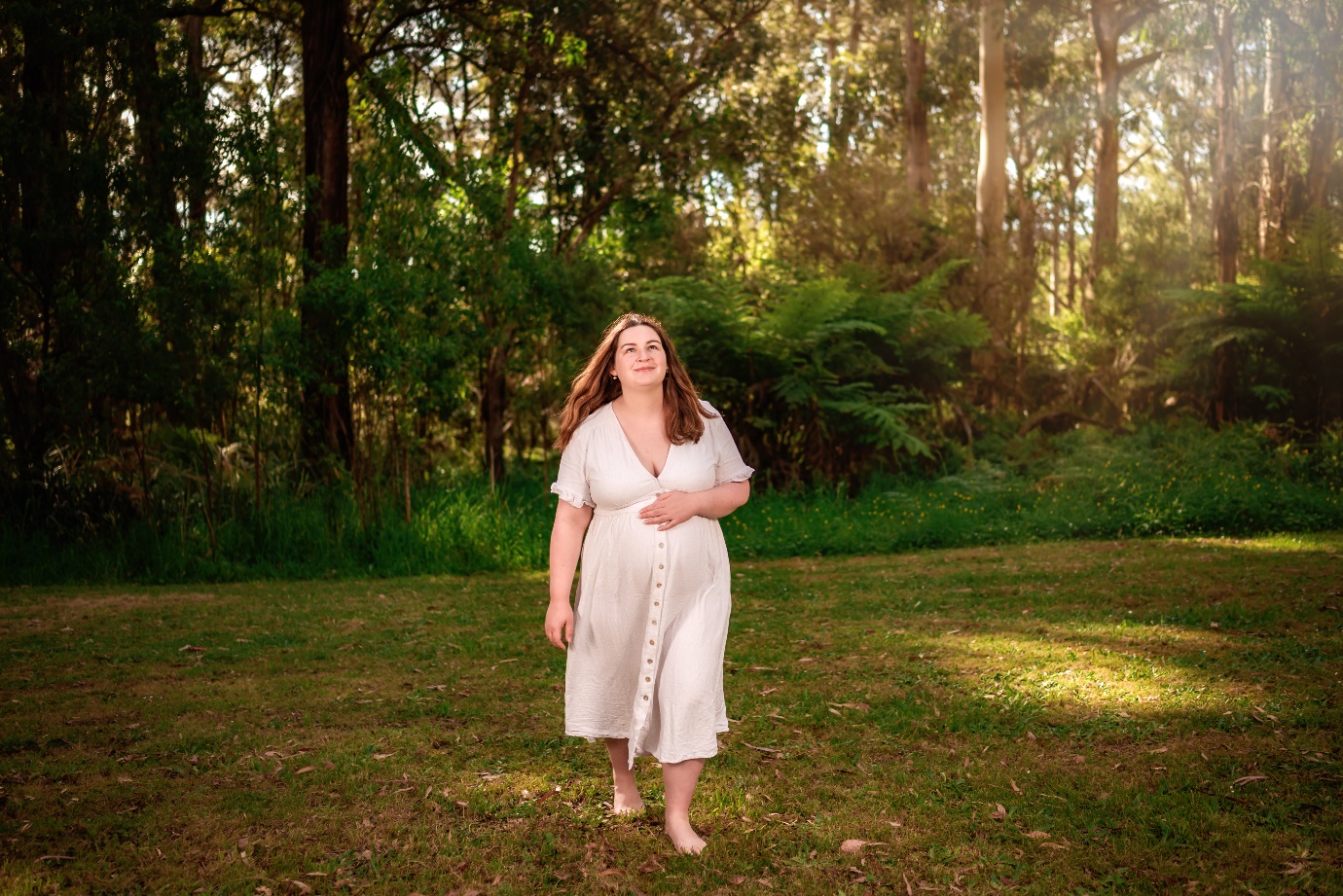
**

**Figure S1d:** Photo of pregnant woman for the digital monitor screen in the clinic waiting room

**
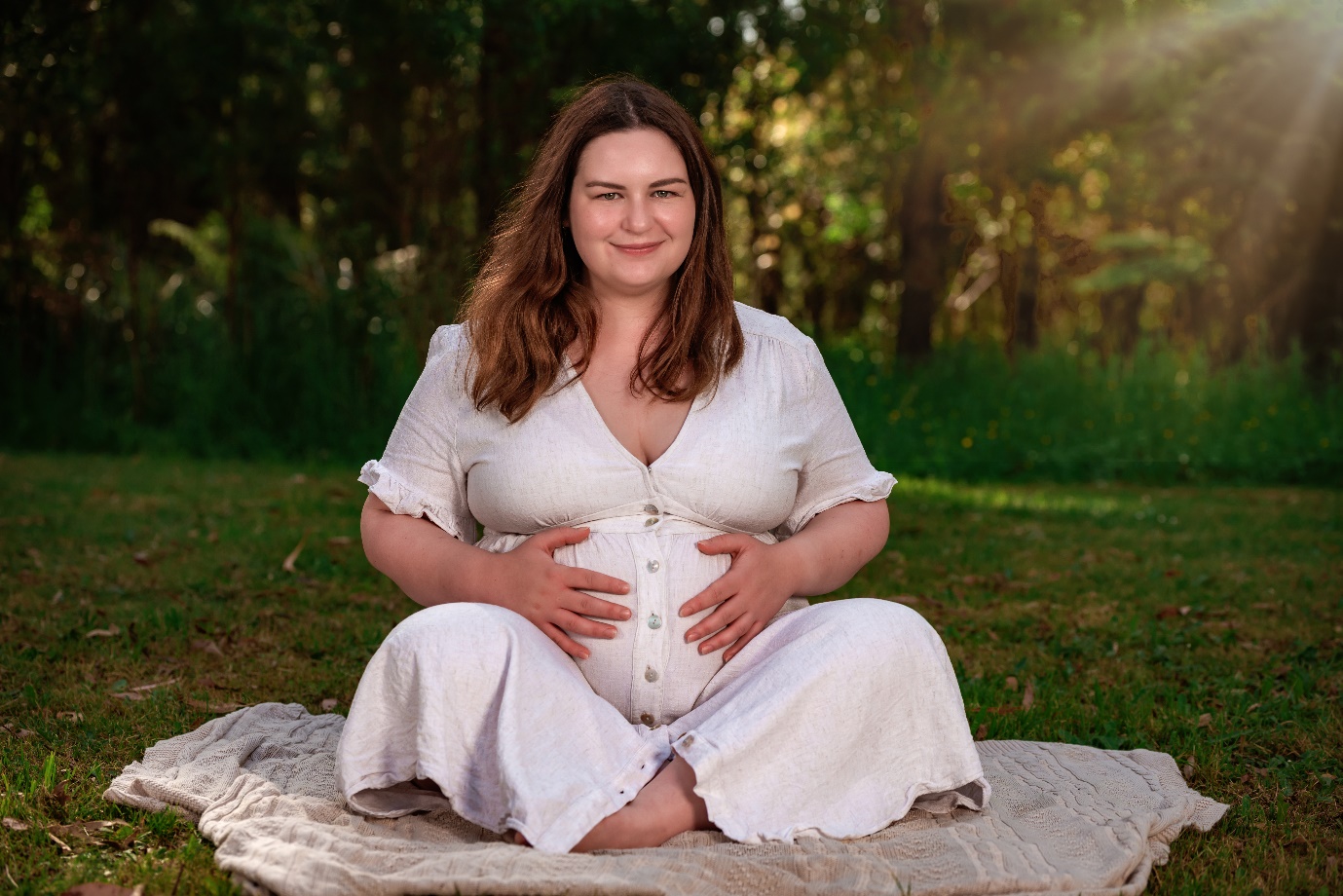
**

**Figure S1e:** Photo of pregnant woman for the digital monitor screen in the clinic waiting room

**
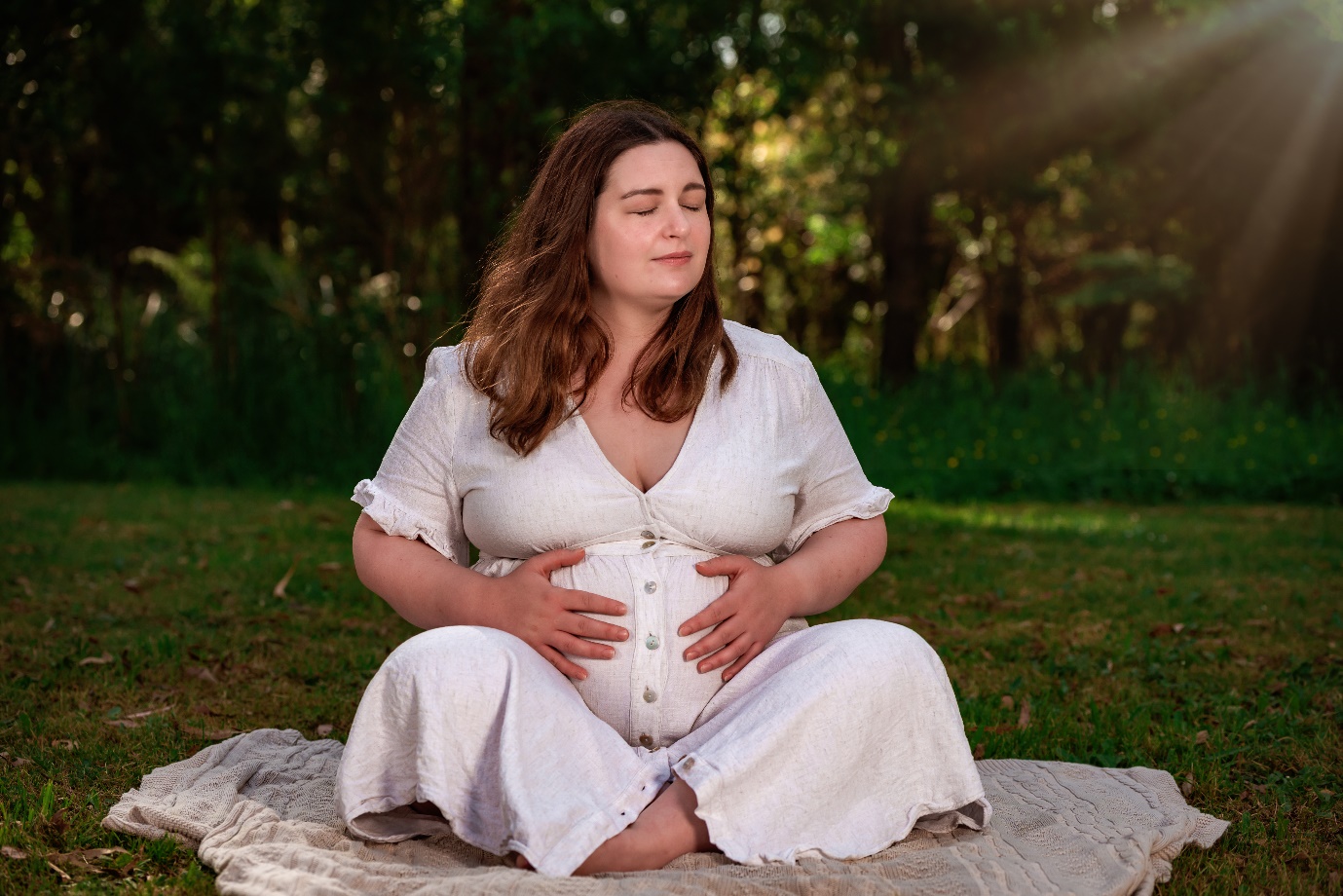
**

**Figure S1f:** Photo of pregnant woman for the digital monitor screen in the clinic waiting room

**
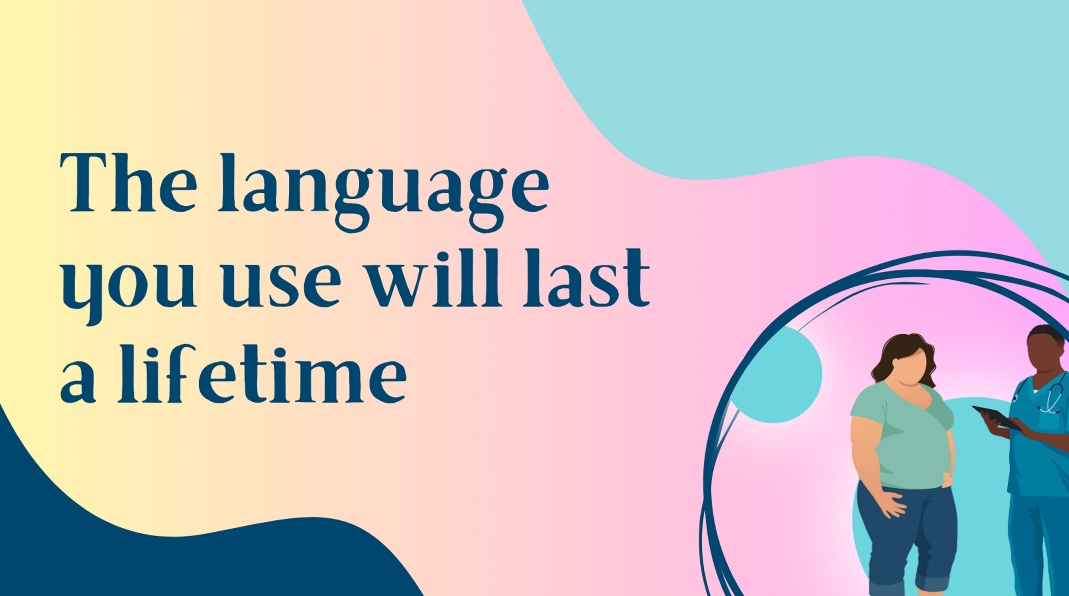
**

**Figure S2a:** Signpost 1 for the antenatal clinic to prompt clinicians to consider weight stigma in their clinical interactions

**
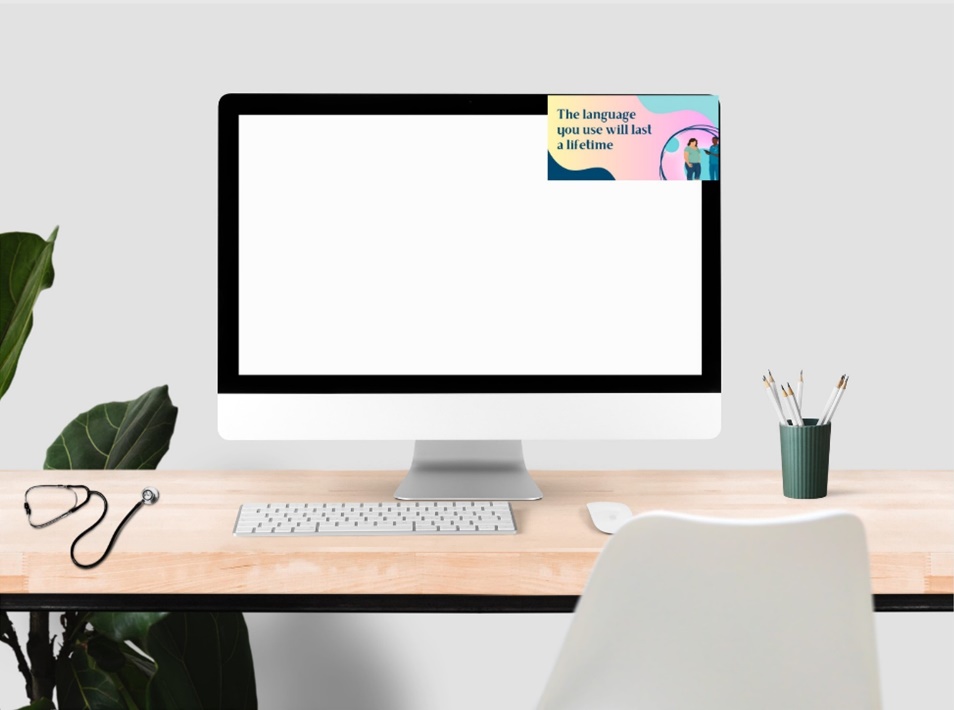
**

**Figure S2b:** Example of signpost 1 placed on a computer screen in the antenatal clinic

**
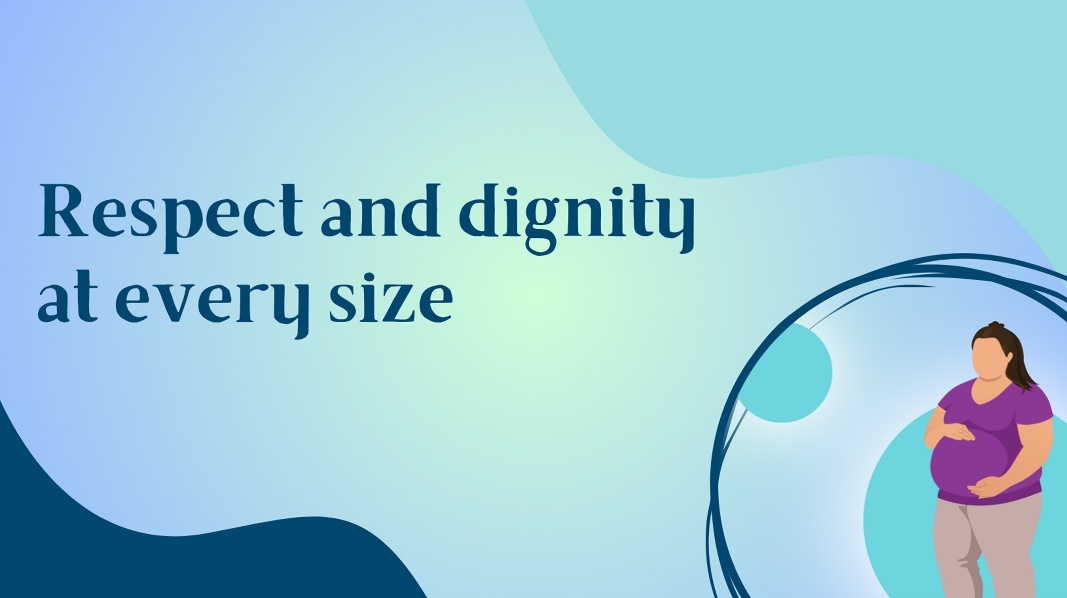
**

**Figure S2c:** Signpost 2 for the antenatal clinic to prompt clinicians to consider weight stigma in their clinical interactions

**
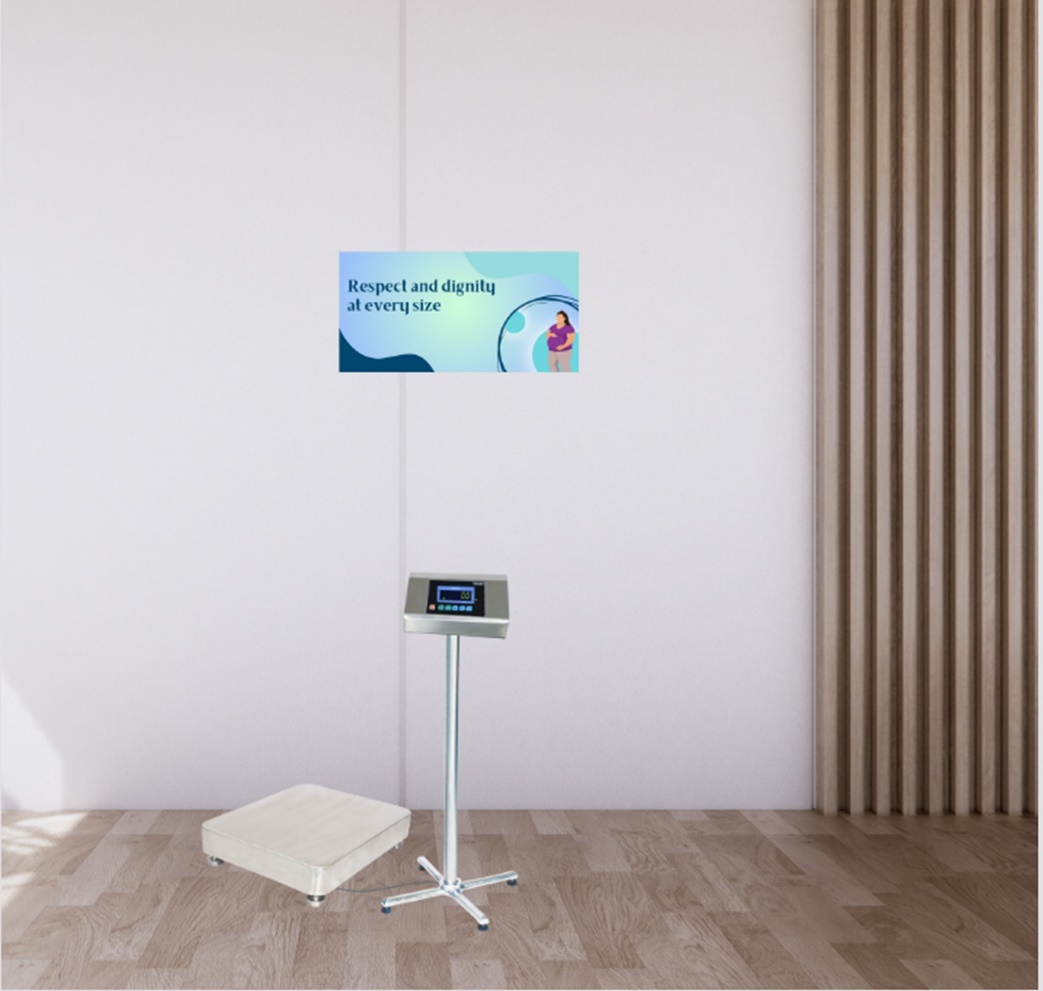
**

**Figure S2d:** Example signpost 2 displayed near the weighing scale in the antenatal clinic

**Table S1. Links to podcast and consumer video**

|  | **Source link** |
| --- | --- |
| Doi link | <https://doi.org/10.17605/OSF.IO/9U52P> |
|  |  |
| URL link | <https://osf.io/9u52p/?view_only=080743973d274964be0df3e429bf8ceb> |

NB: both links take you to the same site to access both the podcast and video

**Table S2. Phase 5: Qualitative interview guide**

| **Phase 5 Qualitative Interview Guide** |
| --- |
| 1. What is your opinion about the relevance and feasibility of these weight stigma resources in reducing weight stigma in ANC practice? |
| 1. What areas do you think these resources do well at? |
| 1. What areas of these resources do you think need improvement? (In terms of acceptability, enhancing awareness etc.) |
| 1. What are your thoughts on the importance the resources in enhancing the motivation of HCPs to reduce weight stigma in their clinical practice? |
| 1. Do you have anything you would like to add? |
